# Comparison of Wrist- and Hip-Worn Activity Monitors When Meeting Step Guidelines

**DOI:** 10.5888/pcd19.210343

**Published:** 2022-04-14

**Authors:** Rachael K. Nelson, Kristina Hasanaj, Gavin Connolly, Laramy Millen, Joshua Muench, Nicole S.C. Bidolli, Michael A. Preston, Alexander H.K. Montoye

**Affiliations:** 1 School of Health Sciences, Central Michigan University, Mount Pleasant, Michigan; 2 College of Health Solutions, Arizona State University, Tempe, Arizona; 3 Department of Nutrition Science, Purdue University, West Lafayette, Indiana; 4 Integrative Physiology and Health Science, Alma College, Alma, Michigan

## Abstract

**Introduction:**

Physical activity (PA) guidelines aimed at accumulating 10,000 steps per day have become increasingly common with the advent of wristband PA monitors. However, accumulated steps measured with wristband PA monitors may not be equal to steps measured with validated, hip-worn pedometers. Consequently, evaluating and developing guidelines for step counts using wristband PA monitors for the general population is needed. We compared step counts accumulated with hip-worn pedometers with those accumulated with wrist-worn activity monitors during 1) treadmill exercise, 2) treadmill walking, and 3) activities of daily living (ADL) to determine their accuracy in meeting step count guidelines (ie, 10,000 steps/d).

**Methods:**

Eighty-six adults (aged 18–65 y; body mass index, 19–45 kg/m^2^) completed 30 minutes of treadmill exercise while simultaneously using a hip-worn pedometer and wrist-worn PA monitor. Remaining steps needed to reach 10,000 steps (ie, 10,000 steps minus the number of pedometer steps recorded from treadmill exercise = remainder) were completed via treadmill walking or ADL. Steps were recorded for both devices after treadmill exercise, treadmill walking, and ADL for both devices.

**Results:**

Fewer steps were accumulated via wrist-worn PA monitors than via hip-worn pedometers during treadmill exercise (3,552 [SD, 63] steps vs 3,790 [SD, 55] steps, *P < .*01) and treadmill walking (5,877 [SD, 83] steps vs 6,243 [SD, 49] steps, *P < .*01). More steps were accumulated via wrist-worn PA monitors than hip-worn pedometers during ADL (7,695 [SD, 207] steps vs 6,309 [SD, 57] steps, *P < .*01). Consequently, total steps were significantly higher for wristband PA monitors than hip-worn pedometers (11,247 [SD, 210] steps vs 10,099 [SD, 39] steps; *P < .*01).

**Conclusion:**

The widely used 10,000-step recommendation may not be accurate for all users of all activity monitors, given the discrepancy in daily step count among wrist-worn and hip-worn devices. Having a more accurate indication of number of steps taken per day based on the device used could have positive effects on health.

**Summary**

**What is known on this topic?**

People commonly use wristband physical activity (PA) monitors to measure steps taken per day so they can accumulate the recommended 10,000 steps. However, wristband PA monitors may not be as accurate in determining the number of steps as validated, hip-worn pedometers.

**What is added by this report?**

We compared the difference between number of steps taken measured using both wrist-worn and hip-worn PA monitors and found significant differences between devices as well as meaningful differences between steps accumulated compared to current PA step guidelines.

**What are the implications for public health practice?**

Recommendations about the number of steps taken per day and messaging around these recommendations with various devices should be re-evaluated in light of these discrepancies.

## Introduction

Engaging in a physically active lifestyle is essential in the prevention and treatment of chronic diseases. While time spent in moderate- or high-intensity activity is the focus of many health organizations (1), completing a certain number of daily steps is also a popular way to encourage physical activity (PA) participation. Step count PA recommendations typically promote the accumulation of 10,000 or more steps per day (2). The goal of accumulating 10,000 steps per day was first introduced in 1965 and thought to be the amount of PA needed to reduce the risk of developing coronary artery disease (3). This recommendation has evolved based on exercise recommendations related to accumulating 30 minutes per day of at least moderate-intensity PA (1,4), PA assessments of free-living individuals (5,6), and recommendations in the newest guidelines aimed at reducing time spent engaged in sedentary behaviors (1). Estimates suggest that most adults engaging in moderate intensity PA accumulate 100 or more steps per minute (4,7). Therefore, evidence supports the notion that 30 minutes of moderate-intensity PA is equivalent to approximately 3,000 steps per day (7). Additionally, free-living individuals who meet the goal of achieving 30 minutes per day of at least moderate-intensity PA accumulate on average 8,000 to 11,000 steps per day (2). Achieving 10,000 steps per day is also associated with meaningful health outcomes (eg, favorable changes to body composition and body mass index [BMI]), even if most steps taken are not at a moderate or higher intensity level (8,9).

Pedometers and, more recently, other wearable devices continue to grow in popularity and are potentially useful for tracking daily step counts and promoting an active lifestyle (10). Wrist-worn monitors, which tend to use triaxial accelerometry, are a more convenient option to track steps and activity than traditional hip- or waist-worn monitors (11). There has been a proliferation of validation studies examining step counts measured by various wrist-worn devices. These studies generally show that wrist-worn activity monitors overestimate steps during free-living conditions by 10% to 35%, underestimate steps during ambulatory activities without wrist movement (eg, pushing a shopping cart, wheelchair, or stroller) by 35% to 95%, and vary in accuracy at different walking speeds (typically underestimating step counts at slower speeds) (12–14). Existing research comparing wrist- and hip-worn devices for step counting in free-living settings generally show that wrist-worn devices overestimate steps by 10% to 25% (12,15–17), sometimes as high as 40% to 50% (18), and sometimes demonstrate no differences between placements (ie, wrist vs hip) (19). This variation in comparability between placements might be due to different monitors used or, more likely, studying populations with different patterns of activity or total activity volume. We are unaware of any previous research comparing device placements for populations meeting step count recommendations (ie, 10,000 steps/d). Given that most of the discrepancy in step counting between wrist and hip devices occurs during nonambulatory activity, it may be that adults who achieve step count recommendations will have less difference in step counts between placement sites due to higher volumes of ambulation.

It remains unclear whether current recommendations of 10,000 steps per day (based on data collected from hip-worn pedometers) is an appropriate target to meet activity guidelines for individuals using wrist-worn monitors or how much discrepancy to expect between wrist- and hip-worn devices to track steps. Therefore, the purpose of this study was to examine steps accumulated on a wrist-worn activity monitor, through treadmill exercise, treadmill walking, and activities of daily living (ADL), compared with a hip-worn pedometer monitor when participants accumulate approximately 10,000 steps per day.

## Methods

A total of 86 men (n = 36) and women (n = 50) (aged 18–65 y; BMI, 19–45 kg/m^2^) completed 2 experimental conditions from September 2016 through June 2019. Three methods were used to recruit participants for this study: 1) a university campus-wide announcement sent out via email, 2) flyers posted around campus and the surrounding area, and 3) word of mouth (eg, participants asking their partners if they would be interested in participating). Participants included normal weight-to-obese adults, including regular exercisers (ie, reported accumulating at least 30 minutes of planned PA, ≥3 d/wk, for the last ≥6 months) and nonexercisers (ie, no planned exercise). All participants were apparently healthy adults (ie, no known cardiovascular or metabolic disease) as assessed via the Physical Activity Readiness Questionnaire (PAR-Q), were nonsmokers, and had no known limiting musculoskeletal or joint issues that would prevent them from engaging in exercise, walking with normal gait, or completing ADL. Written informed consented was obtained from all participants before participation, and all procedures were approved by the Central Michigan University Institutional Review Board.

### Study design

Participants completed an experimental trial where steps were accumulated and recorded from a hip-worn Omron pedometer and wrist-worn Fitbit monitor during 3 modalities: 1) treadmill exercise, 2) treadmill walking, and 3) ADL. Three modalities were used to create 2 experimental conditions. The first experimental condition was a controlled setting in our laboratory where steps were accumulated completely on a treadmill during 30 minutes of treadmill exercise (designed to meet current exercise guidelines) followed by treadmill walking to accumulate the remaining steps needed to reach a total of 10,000 steps. This condition was referred to as *laboratory setting* and allowed for careful control of participants to ensure they took 10,000 steps. The second experimental condition was designed to represent how most US adults might meet exercise and physical activities guidelines. Data from the National Health and Nutrition Examination Survey indicate that walking, bicycling, and ADL (eg, yard work) are the most commonly reported leisure-time physical activities among a representative group of US adults (20). Therefore, in the second experimental condition, steps accumulated through treadmill exercise were combined with steps accumulated through ADL. This experimental condition was referred to as *real-world setting.*

Age of participants was determined by their date of birth reported on the PAR-Q form. After participants completed the PAR-Q form, they were asked if they were regular exercisers or nonexercisers. “Exerciser” was defined as a participant who accumulated at least 30 minutes of planned PA on 3 or more days per week for the last 3 or more months; “nonexerciser” was defined as one who reported no planned exercise. Next, while wearing light clothing and no shoes, participants’ height was determined using a stadiometer (Seca 213, Seca GmbH), and weight was measured using a standard physician scale (Seca 700, Seca GmbH). Participants’ stride length was determined via manufacturer guidelines prior to being fit with the hip-worn pedometer.

### Devices used

Participants were fitted with an Omron HJ-720 ITC pedometer (Omron Health Care, Inc), which was programmed with user weight and stride length. The Omron was worn as the reference device by participants to provide a step count measure during treadmill exercise, treadmill walking, and ADL. According to manufacturer instructions, participants were fitted with the device at the anterior axillary line of the right hip, clipped to the pant waist. Study staff demonstrated and checked for proper placement on the hip. The Omron HJ-720 ITC provides accurate measures of steps taken in laboratory and free-living settings, rendering it a suitable criterion measure for comparison in this study (21,22).

Next, participants were fitted with a Fitbit Charge HR (Fitbit, Inc), worn on the dorsal side of the nondominant wrist (dominant hand was defined as the hand they preferred to write with), per manufacturer recommendations, before data collection. Before study start, the user height, weight, and sex were input into the Fitbit mobile application and synced to the device.

### Treadmill exercise

Treadmill exercise consisted of 30 minutes of exercise on a treadmill at a speed of 1.5–5.8 mph and grade of 1.4%–3.0% to reach 64%–74% of participants’ age-predicted heart rate (HR) maximum (pHRmax = 220 minus age) and equivalent to moderate-intensity exercise (12). During this exercise session, participants wore a Polar chest strap (Polar H7 Heart Rate Sensor, Polar Electro) to monitor HR during exercise. The HR monitor was worn along the rib cage, just below the xiphoid process of the sternum, secured in place with an elastic strap, and transmitted to a Polar watch (Polar A300, Polar Electro) held by the research team. Speed and/or grade were adjusted within the first 5 minutes of exercise only to meet participants’ target HR range (ie, 64%–74% pHRmax). HR was recorded every 5 minutes during the exercise session to determine the average percent HRmax (%HRmax = [average HR/pHRmax] × 100) at which participants exercised during the entire exercise bout. Additionally, oxygen consumption (VO_2_) was estimated based on speed and grade using American College of Sports Medicine equations for treadmill activity (23) and metabolic equivalents (METs), a second indicator of exercise intensity, were calculated (METs = estimated VO_2_/3.5). After exercise was complete, participants immediately stepped onto the sides of the treadmill, and steps from the Omron and Fitbit were recorded. Next, using the Omron as the criterion device, the remaining steps needed to accumulate a total of 10,000 steps were performed via treadmill walking (shortly after the exercise session) and ADL (the following day). Because steps accumulated during treadmill exercise ranged from approximately 3,000 to 5,000 steps (using the Omron), remaining steps to be completed during treadmill walking (to complete the laboratory setting condition) and ADL (to complete the real-world setting condition) ranged from approximately 5,000 to 7,000 steps.

### Treadmill walking

Treadmill walking was performed at 3 mph and 0.5% grade. Again, the Omron was used as a criterion reference; steps were monitored until the Omron reached a total of 10,000 steps from the treadmill exercise and walking conditions. Once these steps were achieved, participants stepped onto the sides of the treadmill; step counts on both devices, total walking time, and total distance covered (reported on the treadmill) were recorded. Step counts accumulated during treadmill exercise and treadmill walking were combined to determine total steps in the laboratory setting for both devices.

### Activities of daily living 

ADL steps were accumulated outside of our laboratory. Participants were sent home with the Omron and Fitbit and instructed to wear both the day after laboratory testing. Participants were shown how to secure each device in the correct orientation and appropriate anatomical location. They were instructed to accumulate their steps through their normal daily activities but not through exercise. Again, the Omron was used as a criterion reference; steps were monitored by participants until they accumulated an amount equal to the number accumulated during the previous day’s treadmill walking. Therefore, participants accumulated 5,000 to 7,000 steps through their ADL. After Omron steps reached the required step count, steps on the Omron and Fitbit were immediately recorded by the participant. Then devices and ADL step count data were returned to the research staff. Steps counts accumulated during treadmill exercise and ADL were combined to determine total steps in the real-world setting scenario for both devices. In total, the 3 PA modalities (ie, treadmill exercise, treadmill walking, and ADL) were completed to create 2 experimental conditions (ie, laboratory setting and real-world setting) to evaluate step count differences between devices. We summed the number of steps accumulated from treadmill exercise and those accumulated from treadmill walking for both devices and subtracted the number of steps accumulated from the Fitbit from those accumulated from the Omron to determine the difference in total steps in the laboratory setting. We summed the number of steps accumulated from treadmill exercise and those accumulated from ADL for both devices and subtracted the number of steps accumulated from the Fitbit from those accumulated from the Omron to determine the difference in total steps in the real-world setting, using the following formulas:

Difference in total steps, laboratory setting = (treadmill exercise + treadmill walking Omron steps) – (treadmill exercise + treadmill walking Fitbit steps)

Difference in total steps, real-world setting = (treadmill exercise + ADL Omron steps) – (treadmill exercise + ADL Fitbit steps)

### Statistical analyses

A paired-samples *t* test was used to assess potential differences between step counts of devices (ie, Omron vs Fitbit) for each condition exercise, walking, ADL, total step counts (laboratory and real-world setting). Bland-Altman statistics were performed to determine the limits of agreement for the Fitbit device compared with the Omron criterion. A subanalysis was performed to determine potential differences between sexes (men vs women) and exercise status (exercisers vs nonexercisers) in step count differences between devices within each condition (ie, treadmill exercise, treadmill walking, and ADL) for each device using an unpaired *t* test or Mann–Whitney rank sum test for nonparametric data. A Pearson correlation was used to assess age and BMI vs difference in total steps between devices. All statistical analyses were performed using SigmaPlot 12.5 (Systat Software, Inc). Significance was set at *P* < *.*05.

## Results

### Participant treadmill exercise and walking characteristics

Participants characteristics are detailed in the Table. During treadmill exercise, participants exercised for 30 minutes at 69.1% (SD, 3.3%) pHRmax, with an average speed of 3.8 (SD, 0.7) mph, a grade of 2.9% (SD, 2.0%), and a distance of 1.9 (SD, 0.3) miles. During treadmill walking, participants walked at 3.0 mph and a 0.5% grade for 55.8 (SD, 5.3) minutes.

**Table:** Characteristics of Participants (N = 86), Study on the Correlation Between Steps Per Day Measured Using Wristband Monitors and Current Step Guidelines, Michigan, US, September 2016–June 2019^a^

Variable	Value	Range
Sex, no.		
Male	36	—
Female	50	—
Age, y	37.7 (14.8)	18–66
Height, cm	171.5 (10.6)	137.2–199.0
Weight, kg	79.0 (17.5)	48.1–123.4
Body mass index, kg/m^2^	26.8 (5.5)	17.7–45.3
Exerciser, %	79	—

Abbreviation: — , not applicable.

a Values are mean (SD) unless otherwise indicated.

### Step counts

**Figure 1:**
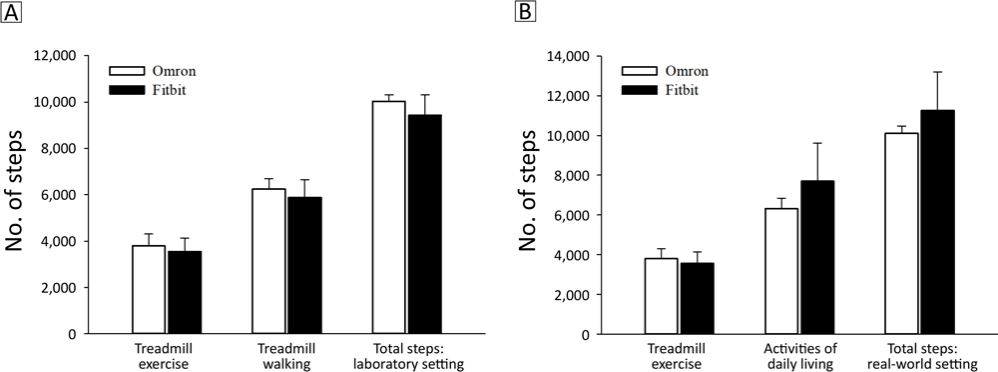
Step counts detected with the Omron pedometer and Fitbit wristband activity monitors during A) treadmill exercise, treadmill walking, and treadmill exercise plus walking combined (total steps: laboratory setting); and B) treadmill exercise, activities of daily living, and treadmill exercise plus activities of daily living (total steps: real-world setting). All measurements significantly different at *P* < *.*05.

**Figure 2:**
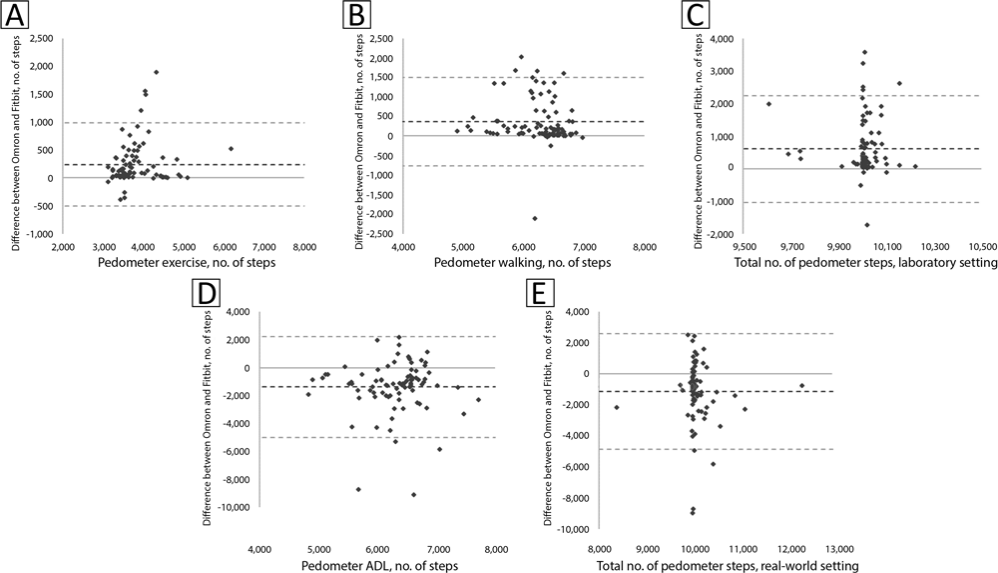
Bland-Altman plots representing differences between Omron pedometer and Fitbit wrist-worn activity monitor steps vs A) Omron pedometer steps during treadmill exercise, B) Omron pedometer steps during treadmill walking, C) total Omron pedometer steps during treadmill exercise plus walking (ie, laboratory setting), D) Omron pedometer steps during activities of daily living (ADL), and E) total Omron pedometer steps during treadmill exercise plus ADL (ie, real-world setting).

Compared with the Omron, significantly fewer steps were accumulated by the Fitbit during walking (*P* < *.*01) (Figure 1) and exercise (*P* < *.*01) (Figure 2). Consequently, when combining steps from treadmill exercise and treadmill walking in the laboratory setting, significantly fewer steps were accumulated with the Fitbit than with the Omron (*P* < *.*01, Figure 1).

**Table:** 

Activity type	Omron pedometer	Fitbit wristband monitor
	Number of steps (standard deviation)	
Laboratory setting		
Treadmill exercise	3,763 (441)	3,529 (533)
Treadmill walking	6,243 (451)	5,871 (767)
Real-world setting		
Treadmill exercise	3,763 (441)	3,529 (533)
Activities of daily living	6,309 (1,850)	7,695 (1,062)

Steps accumulated during ADL with the Fitbit were significantly greater than those from the Omron (*P* < *.*01, Figure 1B). When steps from ADL were combined with steps from treadmill exercise, participants’ total steps in the real-world setting remained significantly higher with the Fitbit compared with the Omron (*P* < *.*01, Figure 1B). Using the Omron as the criterion device, the average difference in total steps between monitors in the laboratory setting was −604 (SD, 838) steps, or a 6.0% difference, and the difference in the real-world setting was 1,148 (SD, 1,898) steps, or 11.4% difference. The larger difference observed for total steps in the real-world setting was driven by a 22% difference between monitors during ADL, compared with a 6% difference during treadmill exercise and a 6% difference during treadmill walking between monitors in the laboratory setting.

Bland-Altman plots are presented in Figure 2 for steps during treadmill exercise (A), walking (B), total steps in the laboratory setting (C), ADL (D), and total steps in the real-world setting (E). Limits of agreement were narrower for the treadmill exercise and treadmill walking conditions (SD, 9.3%–10.0% of criterion steps) than for the ADL condition (SD, 29.3% of criterion steps), resulting in narrow limits of agreement for the total steps in the laboratory setting (SD, 8.3% of criterion steps) but wide limits of agreement for total steps in the real-world setting (SD, 18.8% of criterion steps).

Our subanalysis indicated that the difference in steps between devices was greater for women than men during treadmill exercise (341 [SD, 461] vs 94 [SD, 132] step difference, *P* < *.*001) and walking (567 [SD, 583] vs 87 [SD, 447] step difference, *P* < *.*001), but not ADL (1330 [SD, 1,986] vs 1464 [SD, 1,667] step difference, *P* = .34). No significant differences were found in step discrepancies during treadmill exercise and walking, nor for ADL for exercise and nonexercisers (data not shown). No relationship was observed between either age and difference in total steps (*r* = 0.003, *P* = .69) or BMI and difference in total steps (*r* = 0.19, *P* = .12).

## Discussion

The overall objective of this analysis was to determine potential step differences accumulated through treadmill exercise, treadmill walking, and ADL on a wrist-worn activity monitor versus a hip-worn pedometer with the goal of accumulating 10,000 steps per day. We found that, compared with a hip-worn pedometer, a wrist-worn activity monitor reported fewer steps during treadmill exercise and walking but overreported steps during ADL. We also found that limits of agreement between devices were narrow when steps were accumulated entirely on a treadmill in a laboratory setting, but greater differences and variability between measures were seen during ADL, similar to how steps are accumulated in a real-world setting. These findings suggest that the types of activities engaged in during free living will affect the magnitude of differences between hip- and wrist-worn monitoring devices. Our subanalysis indicated that sex did influence differences between step counts during treadmill activity (ie, exercise and walking) but not during ADL. Additionally, exercise status did not influence step count differences between devices in any modality (treadmill exercise, treadmill walking, or ADL). Finally, we found that participant demographics, including BMI and age, were not associated with the magnitude of step count difference between devices in our real-world setting.

We found that, compared with a hip-worn pedometer, a wrist-worn activity monitor underestimated steps accumulated in a controlled environment (eg, rhythmic activity) during treadmill walking (lower intensity) or exercise (higher intensity). This finding is consistent with previous reports indicating that wrist-worn activity monitors consistently counted fewer steps than hip-worn devices and hand-tallied step counts in controlled environments (eg, laboratory treadmill exercise) (14,24–26). More specifically, we found that the Fitbit Charge underestimated steps by approximately 6%. This finding was similar to previous a report indicating that other Fitbit models also underestimate steps during treadmill walking by approximately 7% (14). In an applied sense, this means that to meet daily target step goals (eg, 10,000 steps/d) on a treadmill, individuals would end up walking more than their original goal when using a wrist-worn Fitbit device.

Consistent with previous reports, we found that wrist-worn devices overestimate steps taken during ADL. Available data indicate that wrist-worn devices overestimate steps accumulated in free-living conditions or during ADL, compared with hip-worn devices (12,26,27). For example, a previous study found that steps from an accelerometer were underestimated with the device worn at the hip and overestimated when the device was worn on the wrist, compared with video observation (28). This finding was attributed to certain activities being considered slower, less repetitive or rhythmic, or less structured, and often involving significant arm movement without corresponding stepping, analogous to ADL in a free-living setting. Conversely, previous work with the hip-worn pedometer used in this study resulted in step underestimations in free-living settings, due to typical household activities usually occurring at slower ambulation speeds (29,30). Collectively this suggests that activity monitor device and modality of activity should be considered when determining step goals.

Differences in steps between devices appeared to be largely due to device location. For example, a nearly 19% overestimation of steps was reported with a wrist-worn Fitbit compared with a hip-worn accelerometer averaged over a 2-day period (12). This discrepancy appears to be more location-specific than device-specific because a similar 17% approximate overestimation of accumulated steps averaged over a 7-day period was reported with an accelerometer worn at the wrist versus the waist (31). These overestimations of steps accumulated with wrist- versus hip-worn devices were slightly higher than the approximate 11% difference we observed in our real-world setting. Although the smaller magnitude of difference we observed may be due to discrepancies in step counts accumulated with waist-worn accelerometers versus pedometers, the potential difference across different types of hip-worn devices appears to be marginal (28). Rather, the smaller magnitude of difference between wrist- and hip-worn devices in our study is more likely the result of PA modality and total activity level. For example, our real-world setting included structured treadmill exercise, producing more consistent step counts between devices than ADL. Additionally, differences in accumulated step counts appear to be greater as step counts per day increase (31). Our study design limited daily steps to 10,000, whereas previous investigations under free-living conditions had a higher range of step counts, especially for more active adults (15,31). Regardless of the magnitude of difference between wrist- and hip-worn devices in various studies, a consistent finding is that wrist-worn devices report more steps than waist/hip-worn devices (ie, 10%–25%). Unfortunately, this fact could lead wrist-worn device users to think that they have met their PA goals for the day when they indeed have not.

Whereas PA modality impacted step counts, demographics including age, BMI, and exercise status had little to no impact on step counts, regardless of device. In fact, BMI status and age were not associated with step differences between devices. Additionally, although we observed greater steps accumulated among participants classified as exercisers compared with nonexercisers during treadmill exercise, exercise status did not influence step count differences between devices. Step count differences between exercisers and nonexercisers during treadmill exercise was most likely due to the greater distance covered during treadmill exercise among exercisers versus nonexercisers (approximately 2 vs 1.75 miles). Interestingly, sex appeared to impact the difference in step counts between devices only during treadmill activity. More specifically, the discrepancy between step counts on devices was greater for female compared with male participants, during treadmill exercise and walking. Female participants in this study were shorter in stature than male participants, potentially resulting in a shorter stride length. This may have contributed to a shorter arm swing that could have been missed by the Fitbit, contributing to the greater discrepancy we observed between the hip and wrist devices for female participants. Overall, this finding suggests that regardless of age, exercise, and BMI status, individuals who engage primarily in walking where arms can swing freely, as opposed to walking where arms could remain relatively stationary (eg, pushing a grocery cart), can aim to achieve 10,000 steps per day irrespective of their monitoring device placement (hip or wrist). However, individuals who mostly perform nonambulatory ADL should aim for 11,000 to 12,000 steps or more per day again regardless of age, exercise, or BMI status if using a wrist-worn monitor.

Our study has limitations. First, while the use of the Omron pedometer is a noted strength (as the gold standard of step measurement), the accuracy of pedometers is dependent on speed and has shown to yield inaccurate step counts at slower ambulation speeds observed during household activities (eg, vacuuming, sweeping) (29,30). Therefore, the Omron may undercount steps in free-living conditions. Additionally, although the wrist-worn activity monitor used (ie, Fitbit Charge) was the most up-to-date at the time of data collection, it has undergone several updates since the time of the study. However, it remains to be a widely used, popular device. Importantly, updated wrist-worn activity monitors have yielded similar results to what we present here (26). More specifically, wrist-worn devices demonstrate decreased accuracy at lower speeds and for nonambulatory movements and in free-living settings (28). Another limitation of our analysis is that it included only 1 day of measurement in a laboratory or real-world setting, which may not represent habitual PA patterns. Future research is needed to better understand the accuracy of various activity monitors regarding habitual PA behavior. However, our study design allowed us to examine conditions of a real-world setting aimed at mimicking free-living patterns in addition to the controlled laboratory setting. Importantly, previous work comparing wrist-worn activity monitors to pedometers has largely included either a free-living or laboratory setting (12,14,24,25,27–31). Furthermore, most existing studies included a small and homogeneous population. A notable strength of this work is the large and diverse sample, increasing the generalizability of findings.

Overall, the often-cited 10,000 steps per day recommendation may not be appropriate for everyone. Several factors should be considered for accurate step recommendations, including type of activity performed, type of activity monitor being used, and location of the activity monitor. Other factors to consider include individuals’ goals. For example, based on a population study, men may require 11,000 to 12,000 steps per day and women 8,000 to 12,000 steps per day to maintain a normal weight (6,23). These considerations are essential to accurately provide daily step recommendations.

In summary, we found that wrist-worn devices overestimate step count in a real-world setting compared with hip-worn pedometers. The results also showed that in a controlled laboratory setting the wrist-worn device underestimated total step count compared with the hip-worn device. The approximate mean step count difference between the wrist- and hip-worn devices was 1,500 steps per day. The widely used 10,000 step recommendation may not be an accurate messaging tool for all activity monitor users, given the discrepancy in daily step count among wrist- and hip-worn devices. More accurately, accumulating daily steps based on the device and placement, type of activity, and PA goals could have positive impacts on health.

## References

[1] Department of Health and Human Services; 2018 Physical Activity Guidelines Advisory Committee. Physical Activity Guidelines Advisory committee scientific report 2018;779 p.

[2] Tudor-Locke C , Craig CL , Brown WJ , Clemes SA , De Cocker K , Giles-Corti B , How many steps/day are enough? For adults. Int J Behav Nutr Phys Act 2011;8(1):79. 10.1186/1479-5868-8-79 21798015 PMC3197470

[3] Bassett DR Jr , Toth LP , LaMunion SR , Crouter SE . Step counting: a review of measurement considerations and health-related applications. Sports Med 2017;47(7):1303–15. 10.1007/s40279-016-0663-1 28005190 PMC5488109

[4] Tudor-Locke C , Sisson SB , Collova T , Lee SM , Swan PD . Pedometer-determined step count guidelines for classifying walking intensity in a young ostensibly healthy population. Can J Appl Physiol 2005;30(6):666–76. 10.1139/h05-147 16485518

[5] Tudor-Locke C , Bassett DR Jr . How many steps/day are enough? Preliminary pedometer indices for public health. Sports Med 2004;34(1):1–8. 10.2165/00007256-200434010-00001 14715035

[6] Tudor-Locke C , Hatano Y , Pangrazi RP , Kang M . Revisiting “how many steps are enough?”. Med Sci Sports Exerc 2008;40(7 Suppl):S537–43. 10.1249/MSS.0b013e31817c7133 18562971

[7] Marshall SJ , Levy SS , Tudor-Locke CE , Kolkhorst FW , Wooten KM , Ji M , Translating physical activity recommendations into a pedometer-based step goal: 3,000 steps in 30 minutes. Am J Prev Med 2009;36(5):410–5. 10.1016/j.amepre.2009.01.021 19362695

[8] Dwyer T , Hosmer D , Hosmer T , Venn AJ , Blizzard CL , Granger RH , The inverse relationship between number of steps per day and obesity in a population-based sample: The AusDiab Study. Int J Obes 2007;31(5):797–804. 10.1038/sj.ijo.0803472 17047641

[9] Tudor-Locke C , Bassett DR Jr , Rutherford WJ , Ainsworth BE , Chan CB , Croteau K , BMI-referenced cut points for pedometer-determined steps per day in adults. J Phys Act Health 2008;5(Suppl 1):S126–39. 10.1123/jpah.5.s1.s126 18364517 PMC2866423

[10] Tudor-Locke C , Lutes L . Why do pedometers work? A reflection upon the factors related to successfully increasing physical activity. Sports Med 2009;39(12):981–93. 10.2165/11319600-000000000-00000 19902981

[11] Adam Noah J , Spierer DK , Gu J , Bronner S . Comparison of steps and energy expenditure assessment in adults of Fitbit Tracker and Ultra to the Actical and indirect calorimetry. J Med Eng Technol 2013;37(7):456–62. 10.3109/03091902.2013.831135 24007317

[12] Degroote L , De Bourdeaudhuij I , Verloigne M , Poppe L , Crombez G . The accuracy of smart devices for measuring physical activity in daily life: validation study. JMIR Mhealth Uhealth 2018;6(12):e10972. 10.2196/10972 30545810 PMC6315254

[13] Chen M-D , Kuo C-C , Pellegrini CA , Hsu M-J . Accuracy of wristband activity monitors during ambulation and activities. Med Sci Sports Exerc 2016;48(10):1942–9. 10.1249/MSS.0000000000000984 27183123

[14] Nelson MB , Kaminsky LA , Dickin DC , Montoye AHK . Validity of consumer-based physical activity monitors for specific activity types. Med Sci Sports Exerc 2016;48(8):1619–28. 10.1249/MSS.0000000000000933 27015387

[15] Chu AHY , Ng SHX , Paknezhad M , Gauterin A , Koh D , Brown MS , Comparison of wrist-worn Fitbit Flex and waist-worn ActiGraph for measuring steps in free-living adults. PLoS One 2017;12(2):e0172535. 10.1371/journal.pone.0172535 28234953 PMC5325470

[16] Gomersall SR , Ng N , Burton NW , Pavey TG , Gilson ND , Brown WJ . Estimating physical activity and sedentary behavior in a free-living context: a pragmatic comparison of consumer-based activity trackers and ActiGraph accelerometry. J Med Internet Res 2016;18(9):e239. 10.2196/jmir.5531 27604226 PMC5031913

[17] Mikkelsen MK , Berg-Beckhoff G , Frederiksen P , Horgan G , O’Driscoll R , Palmeira AL , Estimating physical activity and sedentary behaviour in a free-living environment: a comparative study between Fitbit Charge 2 and Actigraph GT3X. PLoS One 2020;15(6):e0234426. 10.1371/journal.pone.0234426 32525912 PMC7289355

[18] Sushames A , Edwards A , Thompson F , McDermott R , Gebel K . Validity and reliability of Fitbit Flex for step count, moderate to vigorous physical activity and activity energy expenditure. PLoS One 2016;11(9):e0161224. 10.1371/journal.pone.0161224 27589592 PMC5010194

[19] Dominick GM , Winfree KN , Pohlig RT , Papas MA . Physical activity assessment between consumer- and research-grade accelerometers: a comparative study in free-living conditions. JMIR Mhealth Uhealth 2016;4(3):e110. 10.2196/mhealth.6281 27644334 PMC5048058

[20] Dai S , Carroll DD , Watson KB , Paul P , Carlson SA , Fulton JE . Participation in types of physical activities among US adults — National Health and Nutrition Examination Survey 1999–2006. J Phys Act Health 2015;12(Suppl 1):S128–40. 10.1123/jpah.2015-0038 26083795 PMC4487906

[21] Lee JA , Williams SM , Brown DD , Laurson KR . Concurrent validation of the Actigraph gt3x+, Polar Active accelerometer, Omron HJ-720 and Yamax Digiwalker SW-701 pedometer step counts in lab-based and free-living settings. J Sports Sci 2015;33(10):991–1000. 10.1080/02640414.2014.981848 25517396

[22] Holbrook EA , Barreira TV , Kang M . Validity and reliability of Omron pedometers for prescribed and self-paced walking. Med Sci Sports Exerc 2009;41(3):670–4. 10.1249/MSS.0b013e3181886095 19204582

[23] American College of Sports Medicine. Riebe D, Ehrman JK, Liguori G, Magal M. ACSM’s guidelines for exercise testing and prescription. 10th edition. New York (NY): Wolters Kluwer; 2018. 472 p.

[24] Imboden MT , Nelson MB , Kaminsky LA , Montoye AH . Comparison of four Fitbit and Jawbone activity monitors with a research-grade ActiGraph accelerometer for estimating physical activity and energy expenditure. Br J Sports Med 2018;52(13):844–50. 10.1136/bjsports-2016-096990 28483930

[25] Rosenberger ME , Buman MP , Haskell WL , McConnell MV , Carstensen LL . Twenty-four hours of sleep, sedentary behavior, and physical activity with nine wearable devices. Med Sci Sports Exerc 2016;48(3):457–65. 10.1249/MSS.0000000000000778 26484953 PMC4760880

[26] Mendoza AR , Lyden K , Sirard J , Staudenmayer J , Tudor-Locke C , Freedson PS . Step count and sedentary time validation of consumer activity trackers and a pedometer in free-living settings. J Meas Phys Behav 2019;2(2):109–17. 10.1123/jmpb.2018-0035

[27] Ummels D , Beekman E , Theunissen K , Braun S , Beurskens AJ . Counting steps in activities of daily living in people with a chronic disease using nine commercially available fitness trackers: cross-sectional validity study. JMIR Mhealth Uhealth 2018;6(4):e70. 10.2196/mhealth.8524 29610110 PMC5902695

[28] Toth LP , Park S , Springer CM , Feyerabend MD , Steeves JA , Bassett DR . Video-recorded validation of wearable step counters under free-living conditions. Med Sci Sports Exerc 2018;50(6):1315–22. 10.1249/MSS.0000000000001569 29381649

[29] Silcott NA , Bassett DR Jr , Thompson DL , Fitzhugh EC , Steeves JA . Evaluation of the Omron HJ-720ITC pedometer under free-living conditions. Med Sci Sports Exerc 2011;43(9):1791–7. 10.1249/MSS.0b013e318212888c 21311356

[30] Hickey A , John D , Sasaki JE , Mavilia M , Freedson P . Validity of activity monitor step detection is related to movement patterns. J Phys Act Health 2016;13(2):145–53. 10.1123/jpah.2015-0203 26107045

[31] Tudor-Locke C , Barreira TV , Schuna JM Jr . Comparison of step outputs for waist and wrist accelerometer attachment sites. Med Sci Sports Exerc 2015;47(4):839–42. 10.1249/MSS.0000000000000476 25121517

